# Supplementary material for: Characterization of acute effects of football competition on hamstring muscles by muscle functional MRI techniques
Source: PLoS One. 2024 Aug 27;19(8):e0308328. doi: 10.1371/journal.pone.0308328 (PMC11349227; doi:10.1371/journal.pone.0308328)
Supplement: Attachment — Submitted filename: Plos-One-Characterization of acute_Review-vSG02042024.docx [file pone.0308328.s001.docx]

**PLOS ONE Characterization of acute effects of football competition on hamstring muscles by muscle functional MRI techniques**

**Very interesting study. The authors show that the semitendinosus is the most activated hamstring muscle during a soccer match and specifically in its middle and distal portion.**

**Abstract** : Syntax errors « ). »

**Introduction** : a bit too long.

**Materials and methods :**

- **«** three portions for each, but in case of short head of biceps just two portions: proximal and distal). An average of 5 slices were discarded due to motion artifacts » : a limit of the study that has to be discussed.
- The MRI primarily represents the last minutes played. Have all the players played 60 minutes straight without stopping? It's not clear.

**Results**

Ok

**Discussion**

- **«** three portions for each, but in case of short head of biceps just two portions: proximal and distal). An average of 5 slices were discarded due to motion artifacts » : a limit of the study that has to be discussed.
- The MRI primarily represents the last minutes played : to be more discussed.

**Fig 1 and 2** : images quality has to be improved.
