# Supplementary material for: Characterization of acute effects of football competition on hamstring muscles by muscle functional MRI techniques
Source: PLoS One. 2024 Aug 27;19(8):e0308328. doi: 10.1371/journal.pone.0308328 (PMC11349227; doi:10.1371/journal.pone.0308328)
Supplement: Attachment — Submitted filename: Response to reviewers.docx [file pone.0308328.s002.docx]

Journal Requirements:

1. When submitting your revision, we need you to address these additional requirements.Please ensure that your manuscript meets PLOS ONE's style requirements, including those for file naming.

**We have changed the tables and the headings style in order to meet PLOS ONE’s style requirements.**

2. We noticed you have some minor occurrence of overlapping text with the following previous publication(s), which needs to be addressed:

**We have found the overlapping texts you mention. Sorry about that, it has been a terrible mistake. We have changed the sentences in lines 276-278 and 313-314. All changes to the manuscript are written in red.**

In your revision ensure you cite all your sources (including your own works), and quote or rephrase any duplicated text outside the methods section. Further consideration is dependent on these concerns being addressed.

**We have included reference 26. It belongs to our group of researchers.**

3. When completing the data availability statement of the submission form, you indicated that you will make your data available on acceptance. We strongly recommend all authors decide on a data sharing plan before acceptance, as the process can be lengthy and hold up publication timelines. Please note that, though access restrictions are acceptable now, your entire data will need to be made freely accessible if your manuscript is accepted for publication. This policy applies to all data except where public deposition would breach compliance with the protocol approved by your research ethics board. If you are unable to adhere to our open data policy, please kindly revise your statement to explain your reasoning and we will seek the editor's input on an exemption. Please be assured that, once you have provided your new statement, the assessment of your exemption will not hold up the peer review process.

**We have held several meetings between the authors of the study and the Football Club's data protection committee and given that due to the season and data such as age and GPS data each individual can deduce which player he is, we are not allowed to share these dates.**

**Sorry for the error, but we cannot make our data available on acceptance.**

4. We note that Figure 1 includes an image of a patient in the study. 
As per the PLOS ONE policy (<http://journals.plos.org/plosone/s/submission-guidelines#loc-human-subjects-research>) on papers that include identifying, or potentially identifying, information, the individual(s) or parent(s)/guardian(s) must be informed of the terms of the PLOS open-access (CC-BY) license and provide specific permission for publication of these details under the terms of this license. Please download the Consent Form for Publication in a PLOS Journal (<http://journals.plos.org/plosone/s/file?id=8ce6/plos-consent-form-english.pdf>). The signed consent form should not be submitted with the manuscript, but should be securely filed in the individual's case notes. Please amend the methods section and ethics statement of the manuscript to explicitly state that the patient/participant has provided consent for publication: “The individual in this manuscript has given written informed consent (as outlined in PLOS consent form) to publish these case details”.

**We have added the paragraph you indicate to the Methods section, within the ethics statement paragraph (line 133-135).**

5. Please review your reference list to ensure that it is complete and correct. If you have cited papers that have been retracted, please include the rationale for doing so in the manuscript text, or remove these references and replace them with relevant current references. Any changes to the reference list should be mentioned in the rebuttal letter that accompanies your revised manuscript. If you need to cite a retracted article, indicate the article’s retracted status in the References list and also include a citation and full reference for the retraction notice.

**Done**

**Reviewer #1:**

**Thanks for the time and effort taken in evaluating our manuscript and giving us useful suggestions. We sincerely think that the article has improved thanks to your instructions.**

**All changes to the manuscript are written in red.**

1) Abstract : Syntax errors « ). »

**Deleted, thank you.**

2)Introduction : a bit too long.
**We thank the reviewer for pointing this out. We have deleted some sentences (crossed out and in red sentences):**

**Lines 63-64, 78, 87-91, 96-99, 100-102 and 104.**

**We wanted to delve into the origin of the mfMRI technique but it is true that it is information that does not contribute anything to the objective of the study.**

3)Materials and methods :
- « three portions for each, but in case of short head of biceps just two portions: proximal and distal). An average of 5 slices were discarded due to motion artifacts » : a limit of the study that has to be discussed.

**In order to discuss better this limit of the study as you recommended we have added the paragraph lines 348-352.**

- The MRI primarily represents the last minutes played. Have all the players played 60 minutes straight without stopping? It's not clear.

**Yes, totally agree, the MRI primarily represents the last minutes played, but as some authors indicated (Carmon G et al and Mayer JM et al) there is a linear relationship between the increase in T2 relaxation time and the intensity of the exercise previously practiced. The concept of exercise intensity includes accumulated load, therefore all the exercise carried out during the previous hours is a cumulative load. The players played at least 60 minutes of an official football match, that is, 45 minutes of the first half and at least 15 minutes of the second. There was a 15 minute break between the two parts.**

**We have added a paragraph in the discussion (lines 334-338) in order to explain it more clearly, we hope you consider it an improvement.**

4)Results
Ok

**Thank you.**

5)Discussion
- « three portions for each, but in case of short head of biceps just two portions: proximal and distal). An average of 5 slices were discarded due to motion artifacts » : a limit of the study that has to be discussed.

**Yes, it has been commented in the Materials and Methods section. We have added a paragraph in the discussion section (lines 348-352)**

- The MRI primarily represents the last minutes played : to be more discussed.

**Yes, it has been commented in the Materials and Methods section. We have added a paragraph in the discussion section (lines 334-338).**

Fig 1 and 2 : images quality has to be improved.

**We totally agree. As you recommend us we have improved both images.**

**Reviewer #2:**

**Thanks for the time and effort taken in evaluating our manuscript and giving us useful suggestions. We sincerely think that the article has improved thanks to your instructions.**

**All changes to the manuscript are written in red. We have changed the tables and the headings style in order to meet PLOS ONE’s style requirements.**

1) I would have liked to see some more explanation and expounding upon the GPS data as it relates to the study and the findings. It felt that this portion of data collection was auxiliary to the study and not well utilized.

**Thank you for your observation. Our sample is made up of youth players. We know that there are no differences in GPS upload metrics between young people and professionals [Abade et al 2014]. In fact, as shown previously, HMLD, HSR, PL and TD were slightly higher in young people, compared to the professional category [Coppalle et al 2021]. In the Guitart et al study there were similar external GPS load metrics by playing position [Guitart et al]. If we evaluate the HSR as a representative date of the intensity of the match, in the study by Guitart et al the average was 444.41 m, while our result is 461.1 m. Therefore, the goal of the GPS data during the match in our study was to ensure that all players obtained a level of intensity similar to the average of all matches. I already have it.**

**We have added a paragraph explaining this in lines 150-151. We hope you consider it an improvement to the article.**

2)There were found to be some hard to read sentences/sections of the manuscript as it relates to wording and flow. Please check this as well as typing errors found in the discussion section.

**We have deleted some sentences in the introduction that we consider them hard to read. We have corrected some errors in the discussion section. Thank you**

3)Please check your use of references. There may have been some references or potential citation that may have been missed in the introduction section.

**Yes, we have added some references in the introduction (References 9, 10, 11, 12) and in the section where we talk about GPS data (References 26, 27, 28). Thank you.**

4)I feel like the clinical implications as covered could have been more thorough, and the authors could include potential future research ideas or objectives based on their findings. The clinical implications state that the findings support rehab or strengthening of the semitendinosus as a preventative method after a match, however this may be better achieved to perform prior to the next match but not immediately after a match.

**We have added a new clinical implication. It is related to the fact that we have shown that mfMRI is a good method of analyzing muscle activation after a football match. We should include this method in the diagnostic approach to cases of unusual injury patterns. Paragraph 380-384.**

**Through this study we look for the muscle that is most activated during a football match. As we have indicated in the manuscript, due to the type of exercise performed during a match, it would be expected. The preventive program with support rehab or strengthening of the semitendinosus as you indicate would not only be applicable before or after a match, but it should be applied throughout an entire season in training and before the match, to ensure that the hamstring group is correctly coordinated and working in harmony. Paragraph 367-379.**

5)Also, many believe that injury prevention is not a relative term and the term injury reduction is better suited.

**Thank you for your observation. We have changed “preventive” by “injury reduction”. Lines 54 and 369.**
